# Physical Activity Among Adults in Rural Western North Carolina During the COVID-19 Pandemic

**DOI:** 10.5888/pcd19.220112

**Published:** 2022-11-17

**Authors:** Wade L. Creech, Brooke C. Towner, Rebecca A. Battista

**Affiliations:** 1 Appalachian State University, Boone, North Carolina

## Abstract

**Introduction:**

During the COVID-19 pandemic, measures implemented to protect community health may have influenced how and where people engaged in physical activity. In rural communities, access to resources, the environment, and socioeconomic status could play a role in how adults are physically active. Our study examined locations where rural residents of a county in western North Carolina engaged in physical activity early in the COVID-19 pandemic, their reasons for being physically active, and their perceptions of benefits and barriers related to engaging in physical activity.

**Methods:**

Rural adults (N =297) completed an online survey from August 3 through September 15, 2020, describing their physical activity during the summer of 2020. Data were analyzed using nonparametric measures.

**Results:**

Survey respondents frequently engaged in physical activity in the home (57.8%), at parks or on trails (45.3%), and around their neighborhood (39.4%). The most common types of physical activities at parks or on trails were walking and hiking (99.5%). Across all locations, the most frequently reported reasons for engaging in physical activity were getting out of the house, maintaining fitness and mental health, and exercising.

**Conclusion:**

Our study showed many locations where rural residents were physically active and their reasons for participating in physical activity during the pandemic. Data about perceived benefits of and barriers to physical activity during the pandemic can assist in meeting the current need to increase physical activity levels in rural communities.

**Summary**

**What is already known on this topic?**

Physical activity could be important to physical and mental health during the COVID-19 pandemic. Rural communities often lack access to locations and facilities for physical activity.

**What is added by this report?**

We sought to learn about the locations, reasons, barriers, and benefits related to physical activity in rural areas to better understand the distinct needs of rural communities compared to nonrural communities.

**What are the implications for public health practice?**

Our research may inform physical activity education and the development of physical activity infrastructure in rural communities, thereby improving residents’ levels of physical activity and overall health and wellness.

## Introduction

Physical activity yields both physical and mental health benefits (1). These benefits became even more critical during the COVID-19 pandemic (2,3). However, in the early part of the pandemic, government stay-at-home orders (4) resulted in closure of many common locations for physical activity (eg, fitness centers, parks, trails), which led to a decline in physical activity among many adults (5–8). As a result, people had to adapt their physical activity behaviors.

Walking paths, trails, sidewalks, parks, and recreation facilities are common locations for physical activity (9). Access to such places is an important factor (10) and influences how and whether residents are physically active (7,11). For example, rural residents often lack developed neighborhoods (eg, sidewalks, safe streets) (7), and the pandemic may have restricted access to or closures of trails and parks.

Respondents to recent surveys during the COVID-19 pandemic reported various reasons for engaging in physical activity and various perceptions of benefits. These included having more time available, schedule flexibility, an increased desire to be outside, wanting to decrease stress or anxiety, and other health-related concerns (6,12). Additionally, outdoor activity became especially desirable (5,13) because it facilitated social distancing (12). At the same time, concerns about exposure to COVID-19, lack of available exercise equipment, state or local restrictions, and living conditions were barriers to being physically active (7,8), as were reported “worry/stress” and “lack of motivation” (6).

In 2017, an estimated 1 in 5 people in the US lived in a rural area (14). Although the sparse population of these areas facilitated social distancing, lack of resources such as sidewalks, parks, and fitness clubs may have served as barriers to regular physical activity (10). Although urban and suburban neighborhoods may have such resources (5), rural areas may not (10). To fully interpret the needs of subgroups of rural respondents to national surveys, a full understanding of their available physical activity resources is needed.

The primary objective of our study was to examine the types of available physical activity resources in rural areas and the reasons adults in these areas engaged in physical activity early in the COVID-19 pandemic. A secondary objective was to describe the benefits and barriers to physical activity residents encountered. This information could be useful to public health professionals and clinicians and to land use and public recreation planners in developing an infrastructure to facilitate outdoor physical activity in rural areas (15).

## Methods

An online survey completed from August 3 through September 15, 2020, appraised locations, reasons for physical activity during the COVID-19 pandemic, and related benefits and barriers.

### Participants and recruitment

By using convenience sampling, we recruited from the general adult population of a western North Carolina county with a rural–urban continuum code (RUCC) of 5 and a nonmetropolitan–nonurban population of 20,000 or more that was not adjacent to a metropolitan area (16). Our inclusion criteria were adults aged 18 years or older, residence in rural western North Carolina, the ability to read English, and access to an electronic device to complete the survey. We contacted prospective participants electronically by using listservs from the county health department, 7 public kindergarten through 8th grade schools, 1 public high school, and 3 family-based homeschool organizations. To obtain the lists, we contacted public school administrators, the health department, and local homeschool organizations via email to explain the purpose of our study and to ask them to participate by distributing our survey to their constituents. Using these listservs allowed us to select names from our county of interest. The county health department list included names from programs for people of low socioeconomic status, which allowed us to reach that audience, and homeschool programs let us reach people with children who did not attend public schools. Those who agreed to distribute the survey received an email describing the study and an email message for them to distribute to their listserv along with a link to the online survey (Qualtrics© XM survey software system). Of the approximately 4,500 people we reached through these listservs, 337 met our criteria and agreed to participate after reviewing the survey’s online consent page describing the study's procedures, risks, and benefits. The institutional review board of Appalachian State University determined this study was exempt.

Participants completed the survey from August 3 through September 15, 2020, during phase 2 of pandemic restrictions, which in North Carolina consisted of face mask requirements and limited gatherings (maximum 10 people indoors and 25 people outdoors). Stay-at-home orders were lifted during this period. The survey consisted of 50 questions and took approximately 30 minutes to complete. After completing the survey, participants had the option of entering a drawing for one of 150 $25 gift cards.

### Measures

**Locations for physical activity**. To determine where participants engaged in physical activity, we asked “Please tell us how often you used the location for physical activity during the COVID-19 pandemic,” defined as the current COVID-19 phase 2 in North Carolina. Options were parks and trails, neighborhoods, and in or around the home. Answers were given on a 3–point Likert scale of 1, frequently; 2, sometimes; and 3, never used for physical activity.

**Types of physical activity.** To assess types of activities for these locations, participants were asked, “During the COVID-19 pandemic, what physical activities did you do?” Participants selected all types of physical activity they participated in at each location. Answers for parks and trails and for neighborhood were walking or hiking, jogging or running, biking, playing with kids, sports activities, kayaking or canoeing, and swimming; for home, answers were indoor housework, workouts or exercise, playing in the yard, outdoor sports or games, yard work, and gardening. The 2018 Physical Activity Guidelines for Americans included many of these activities as examples of moderate to vigorous aerobic activities (1).

**Reasons for physical activity.** Participants were asked “Please choose your reason(s) for participating in physical activity” at each location. Response options were as follows: for exercise, to experience nature, to get out of the house, to do something normal, for mental health, to take kids outside, to walk the dog, to connect with others, to maintain health and fitness, to prepare for future events, and to reduce sitting time.

**Benefits and barriers.** Participants were asked to rate their beliefs about the benefits of physical activity and barriers to engaging in it on a Likert scale of 1 to 5, with 1 indicating strongly disagree and 5 indicating strongly agree. The following were benefit options: something I can do without being exposed to COVID-19, offers a routine or something normal, helps manage stress, helps manage mental health, helps manage chronic disease, supports my immune system, reduces the risk of COVID-19, and will reduce the severity of COVID-19. Barrier options were the following: appropriate social distancing, closures of park and recreation resources, cancellation of sport/intramurals, neighborhood environment, closure of fitness facility, lack of fitness equipment, and stay-at-home orders.

**Demographics.** Participants reported their gender (male, female, other), age (categorized as 18–25 y, 26–35 y, 36–45 y, 46–55 y, 56–65 y, ≥66 y), race, education level, annual household income (≤$20,000, $20,000– $59,999, $60,000– $99,999, $100,000– $149,999, ≥$150,000), and number of children aged 18 years or younger in the home (0, 1, 2, 3, 4, ≥5).

Analyses were conducted in 2021 by using SPSS Statistics 27 (IBM Corp) with nonparametric measures. We ran frequencies for demographic information and subjective variables with single-response items. Responses were grouped by the location of physical activity (parks or trails, neighborhood, home) during the pandemic. The percentage of respondents who participated in various activities and the reason for participation were determined by the location of the activity.

## Results

Of the 337 people who agreed to take the survey, 297 completed at least 50% and were included in our sample. Most participants were middle-aged (36–55 y), White, and female (Table 1). Approximately 74.4 % of participants held a post–secondary-education degree (eg, Bachelor, Master, Doctorate, or other professional degree). Participants’ household income varied; 53.9% of earned from $60,000 to $150,000 annually.

**Table 1:** Demographic Characteristics, Adults (N = 297) in Rural North Carolina During the COVID-19 Pandemic, August 3–September 15, 2020^a^

Characteristic	n (%)
Gender	
Male	43 (14.5)
Female	254 (85.5)
Other	1 (0.3)
Age, y	
18–25	6 (2.0)
26–35	33 (11.1)
36–45	110 (37.0)
46–55	104 (35.0)
56–65	34 (11.4)
66–75	10 (3.4)
Race	
American Indian or Alaska Native	3 (1.0)
Asian	8 (2.7)
Black or African American	2 (0.7)
White	285 (96.0)
Annual household income, $	
<20,000	8 (2.7)
20,000–59,999	67 (22.6)
60,000–99,999	92 (31.0)
100,000–149,999	68 (22.9)
≥150,000 or more	35 (11.8)
Prefer not to respond	28 (9.4)
Education	
High school graduate	12 (4.0)
Some college	62 (20.9)
Bachelor’s degree	103 (34.7)
Master’s degree	93 (31.3)
Doctorate/professional degree	25 (8.4)
Prefer not to respond	2 (0.7)

a Values are number (percentage). Percentages may not total 100 because of rounding.

More than half (57.8%) of participants frequently used their homes for physical activity, 39.7% used their homes sometimes, and 2.5% never used their homes. We saw similar numbers in the use of parks and neighborhoods: 45.3% frequently used parks, and 39.4% frequently used their neighborhood; 36% sometimes used parks and 43.2% sometimes used their neighborhood; 18.6% never used parks and 17.4% never used their neighborhood.

The most prevalent types of physical activity in the home were indoor housework (67.8%), yard work/landscaping (60.6%), and workouts/exercise (57.2%) (Table 2). The most prevalent reason for physical activity at home was to maintain health and fitness (76.0%). Other common reasons were for mental health (74.5%) and exercise (66.3%) (Table 3).

**Table 2:** Type of Physical Activity, by Location, Among Adults (N = 297) in Rural North Carolina During the COVID-19 Pandemic, August 3–September 15, 2020^a^

Variable	Parks/trails, n = 183	Neighborhood, n = 176	Home, n = 208
Walking/hiking	182 (99.5)	173 (98.3)	—
Jogging/running	74 (40.4)	50 (28.4)	—
Biking	70 (38.3)	64 (36.4)	—
Playing with kids	82 (44.8)	—	—
Sports	29 (15.8)	29 (16.5)	—
Kayaking/canoeing	79 (43.2)	—	—
Swimming	47 (25.7)	—	—
Indoor housework	—	—	141 (67.8)
Workouts/exercise	—	—	119 (57.2)
Playing in the yard	—	—	116 (55.8)
Outdoor sports/games	—	—	84 (40.4)
Yard work/landscaping	—	—	126 (60.6)
Gardening	—	—	108 (51.9)

Abbreviation: —, Variable was not offered as a response for the listed location.

a Values are number (percentage).

**Table 3:** Reason for Physical Activity, by Location, Among Adults (N = 297) in Rural North Carolina During the COVID-19 Pandemic, August 3–September 15, 2020^a^

Variable	Parks/trails, n = 183	Neighborhood, n = 183	Home, n = 208
For exercise	162 (88.5)	153 (83.6)	138 (66.3)
To experience nature	141 (77.0)	105 (57.4)	—
To get out of the house	174 (95.1)	163 (89.1)	—
To do something normal	123 (67.2)	106 (57.9)	125 (60.1)
For mental health	146 (79.8)	129 (70.5)	155 (74.5)
To take the kids outside	113 (61.7)	107 (58.5)	99 (47.6)
To walk the dog	92 (50.3)	106 (57.9)	—
To connect with others	46 (25.1)	44 (24.0)	28 (13.5)
To maintain health/fitness	139 (76.0)	113 (61.7)	158 (76.0)
To prepare for future events	13 (7.1)	9 (4.9)	17 (8.2)
To reduce sitting time	124 (67.8)	113 (61.7)	134 (64.4)

Abbreviation: —, Variable was not offered as a response for the listed location.

a Values are number (percentage).

Among the 183 respondents who said they used parks and trails, 99.5% used them for walking and hiking. Other popular types of physical activity were playing with kids (44.8%), kayaking or canoeing (43.2%), and jogging or running (40.4%) (Table 2). The most frequent reasons given for participating in physical activity at parks or trails were to get out of the house (95.1%) and for exercise (88.5%). Other reasons were for mental health (79.8%), to experience nature (77.0%), and to maintain health and fitness (76.0%) (Table 3).

For physical activity in neighborhoods, 176 participants reported engaging in some form. Walking and hiking were the most prevalent types (98.3%) followed by bicycling (36.4%) and jogging or running (28.4%) (Table 2). The most prevalent reasons given for physical activity in neighborhoods were to get out of the house (89.1%), for exercise (83.6%), and for mental health (70.5%) (Table 3).

Perceived barriers to and benefits of physical activity varied (Table 4). Respondents either agreed (38.9%) or strongly agreed (33.0%) that closure of parks and recreation resources was a barrier to physical activity. The second leading barrier to participation was stay-at-home orders (26.6% agreed and 29.1% strongly agreed). Respondents strongly agreed about other barriers to physical activity, including the cancellation of recreational sports or intramural games (34.0%) and the closure of fitness facilities (28.6%). More than half of respondents strongly agreed that physical activity helped to manage mental health (56.2%) and stress (53.7%); almost half (46.8%) strongly agreed that physical activity was beneficial to the immune system during the COVID-19 pandemic.

**Table 4:** Perceived Barriers to Physical Activity Among Adults (N = 203) in Rural North Carolina During the COVID-19 Pandemic, August 3–September 15, 2020^a^

Response	Strongly disagree	Disagree	Neither agree or disagree	Agree	Strongly agree
**Barriers**					
Ability to maintain appropriate social distancing	44 (21.7)	48 (23.6)	27 (13.3)	51 (25.1)	30 (14.8)
Closure of parks and recreation resources	13 (6.4)	21 (10.3)	23 (11.3)	79 (38.9)	67 (33.0)
Cancellation of sports or intramurals	45 (22.2)	13 (6.4)	46 (22.7)	30 (14.8)	69 (34.0)
Neighborhood environment	77 (37.9)	50 (24.6)	44 (21.7)	21 (10.3)	11 (5.4)
Closure of fitness facility	41 (20.2)	12 (5.9)	57 (28.1)	33 (16.3)	58 (28.6)
Lack of fitness equipment	45 (22.2)	36 (17.7)	47 (23.2)	43 (21.2)	32 (15.8)
Stay-at-home orders	30 (14.8)	39 (19.2)	33 (16.3)	54 (26.6)	49 (29.1)
**Benefits**					
Is something I can do to leave the house without being exposed to COVID-19	8 (3.9)	11 (5.4)	26 (12.8)	93 (45.8)	65 (32.0)
Is something routine or normal that I can still do while social distancing	6 (3.0)	9 (4.4)	13 (6.4)	104 (51.2)	71 (35.0)
Helps manage stress during the COVID-19 pandemic	3 (1.5)	2 (1.0)	6 (3.0)	83 (40.9)	109 (53.7)
Helps manage my mental health (eg, stress, anxiety) during the COVID-19 pandemic	1 (0.5)	1 (0.5)	6 (3.0)	79 (38.9)	114 (56.2)
Helps me manage my chronic condition(s) (eg, diabetes, hypertension, heart disease)	8 (3.9)	3 (1.5)	78 (138.4)	60 (29.6)	48 (23.6)
Is beneficial to my immune system during the COVID-19 pandemic	3 (1.5)	0 (0)	13 (6.4)	92 (45.3)	95 (46.8)
Will help reduce my risk of getting COVID-19	12 (5.9)	17 (8.4)	64 (31.5)	53 (26.1)	56 (27.6)
Will reduce the severity of COVID-19 if I get it	8 (3.9)	15 (7.4)	63 (31.0)	65 (32.0)	51 (25.1)

a Number reflects survey attrition. Values are number (percentage).

## Discussion

Our evaluation showed that adults living in rural western North Carolina primarily used the home for physical activity in the early COVID-19 pandemic to maintain fitness and mental health. Activities in and around the home were indoor housework, yardwork, and workout or exercise. This finding was similar to other surveys administered during the pandemic in which the home or garage were the most commonly reported locations (5,7). However, our results differed from others in that the second most common location was parks and trails rather than neighborhoods (5,7). Participants in our study gave reasons for using these locations as “to get out of the house,” “for exercise,” and “for mental health.” Our participants also reported going outside to be active and to “do something normal.” Because rural communities often do not have the infrastructure of sidewalks, safe streets, greenways, and playing fields, which are more common in nonrural communities, parks and trails may have been more accessible to them during the pandemic, especially during the stay-at-home phase. Although some of these locations experienced closures during the initial pandemic wave, our data suggest that these spaces were some of the few locations available for physical activity in rural communities.

 Our results also showed that various perceived barriers and benefits to physical activity existed and contributed to use of the locations chosen and why. Because of enforced public health regulations, many people could not engage in their preferred recreational activities (eg, travel, eating out at restaurants, going to movies, using fitness centers) (15). Our collected data support this conclusion, because prominent barriers to physical activity were the closure of fitness facilities and because stay-at-home orders made outdoor locations such as parks and trails and neighborhoods increasingly popular. The increase in outdoor recreation and park use was potentially the result of a recreation substitution, as new outdoor recreationists either tried outdoor recreation for the first time or returned to outdoor recreation after a prolonged hiatus (15).

The benefits of engaging in physical activity in our sample included managing overall health. Our participants reported “helps manage my mental health,” “helps manage stress,” and “is beneficial to my immune system” as reasons. Similarly, others reported stress as a reason for changing health behaviors such as becoming physically active. One study found adults who positively changed health behavior by increasing physical activity during the COVID-19 pandemic did so because they had more time available and used activity to relieve stress and for other health-related concerns (7).

The disparate effects of the COVID-19 pandemic on physical activity levels of potentially vulnerable subgroups, including people with low incomes or who live in rural communities, underscore the need for population-specific physical activity programs and policies over the next several months to years as the pandemic continues (5). Recent evidence from similar studies supports the need for further understanding of subgroups within a population, assessments of their resource concerns, and more information about their physical activity choices to better determine ways to increase physical activity (5,7,8). Although rural communities may lack infrastructure, our results point to the potential use of parks and trails as immediate resources for physical activity. Young adults (18–29 y) engaged in physical activity at parks and open spaces during the early part of the pandemic and continued to be active at these locations 13 months later (13). Similar results suggest the outdoors as an option for future behavioral interventions (17).

Our study had limitations. First, study participants were mainly female, middle-aged, White, with an annual income above $60,000, and a post–secondary-education degree. The lack of diversity reflects the demographics of rural western North Carolina. Other participants in other studies had similar demographic characteristics: a high percentage of females (5,6,8), non-Hispanic people, and residents with post-secondary education (5). According to the Western North Carolina Health Network, the racial and ethnic population of western North Carolina is 89.9% White, 4.3% Black or African American, 1.5% American Indian/Alaska Native, and 6.0% Hispanic or Latino (of any race) (18); 50.2% of the population in the county we studied were female (19). This lack of diversity in our sample is a possible limitation in generalizing results on a broader scale. In addition, our sample included a disproportionately large number of participants with a post–secondary-education degree. In 2019, approximately 35% of Americans held some type of post-secondary academic degree (20), whereas in our sample 74.4% of participants (n = 221) held a post-secondary degree. Thus, education level may influence regular exercise, with the findings showing that people with advanced degrees were more likely to exercise regularly (21). Another limitation was that we developed this survey urgently to address the immediate need for information related to the COVID-19 pandemic. We did not test the survey’s validity or reliability before administering it.

Our study results suggest many locations and reasons for participating in physical activity in rural western North Carolina that could be applied to other rural areas of the US. Perceived barriers and benefits related to physical activity can aid further understanding of why participants engaged in physical activity during the COVID-19 pandemic. Because use of parks and trails contributed to outdoor physical activity among rural residents, access to such spaces is key to increasing and maintaining levels of physical activity after the threat of the COVID-19 pandemic subsides. Understanding where adults want to be physically active may also be helpful when developing interventions and resources (22). The early phases of the COVID-19 pandemic (eg, shelter-in-place, stay-at-home) affected many health-related behaviors and forced adults to change where and why they engaged in physical activity. However, much can be learned from behaviors during this time frame. In the future, public health agencies could assist in creating awareness of available physical activity locations.
